# Restoration of the immune function as a complementary strategy to treat Chronic Lymphocytic Leukemia effectively

**DOI:** 10.1186/s13046-021-02115-1

**Published:** 2021-10-15

**Authors:** Carol Moreno, Cecilia Muñoz, María José Terol, José-Ángel Hernández-Rivas, Miguel Villanueva

**Affiliations:** 1 Hospital Santa Creu i San Pau, Barcelona, Spain; 2 Hospital Universitario de la Princesa, Madrid, Spain; 3 Hospital Clínico de Valencia, Valencia, Spain; 4 Hospital Universitario Infanta Leonor, Universidad Complutense de Madrid, Madrid, Spain; 5 Servicio de Hematología y Hemoterapia, Hospital Universitario Infanta Leonor, Departamento de Medicina, Universidad Complutense de Madrid, Madrid, España; 6 C/ Gran Vía del Este 80, 28031 Madrid, Spain; 7 Departamento Médico – Hematología, Janssen-Cilag, S.A Spain

**Keywords:** Immune function, Ibrutinib, CLL, Chronic Lymphocytic Leukemia

## Abstract

Chronic Lymphocytic Leukemia (CLL) is a hematological malignancy characterized by uncontrolled proliferation of B-cells and severe immune dysfunction. Chemo(immuno)therapies (CIT) have traditionally aimed to reduce tumor burden without fully understanding their effects on the immune system. As a consequence, CIT are usually associated with higher risk of infections, secondary neoplasms and autoimmune disorders. A better understanding of the biology of the disease has led to the development of therapeutic strategies which not only act against malignant B-cells but also reactivate and enhance the patient’s own anti-tumor immune response. Here, we review the current understanding of the underlying interplay between the malignant cells and non-malignant immune cells that may promote tumor survival and proliferation. In addition, we review the available evidence on how different treatment options for CLL including CIT regimens, small molecular inhibitors (i.e, BTK inhibitors, PI3K inhibitors, BCL-2 inhibitors) and T-cell therapies, affect the immune system and their clinical consequences. Finally, we propose that a dual therapeutic approach, acting directly against malignant B-cells and restoring the immune function is clinically relevant and should be considered when developing future strategies to treat patients with CLL.

## Background

Chronic Lymphocytic Leukemia (CLL) is the most common B-cell malignancy in the Western world [1]. CLL is a lymphoproliferative disease characterized by the accumulation of mature monoclonal B-cells with a typical immunophenotype (i.e, CD5+CD23+ and other B-cell markers) which accumulate in peripheral blood, bone marrow and lymph nodes. A hallmark of the pathophysiology of CLL is the dysfunction of the immune system which is mostly translated in a state of humoral and cellular immunodeficiency and higher prevalence of autoimmune disorders [2, 3].

In CLL, tumor cells influence the immune system to escape immunosurveillance and create an immunosuppressive microenvironment. In general, there are four mechanisms used by CLL cells to escape from the control of immune cells: (1) non-immunogenic tumor cell death; (2) expansion and recruitment of immunosuppressive cells, including T regulatory (T-reg) cells, M2 macrophages and myeloid-derived suppressor cells (MDSC); (3) depletion and/or inhibition of antitumor immune cells such as Th1 T-cells and CD8+ cells; and (4) production of immunosuppressive soluble factors such as IL-10 and TGF-β [4]. These changes are highly relevant for the maintenance and progression of the tumor and contribute to the complex manifestations of the disease. Recently, it has been reported that immune microenvironment plays a significant role in progression of the disease in contrast to clonal evolution [5].

At the end of the 90´s, the introduction of chemotherapy in combination with anti-CD20 monoclonal antibodies such as rituximab was an important step forward in CLL therapy and the outcome of patients significantly improved. However, most patients will eventually relapse. Importantly, CIT decreases the disease burden but, at the same time, exacerbates certain defects of an already dysfunctional immune system which further impacts patients’ health, with increased infection morbidity and secondary neoplasms [6]. The introduction of small molecule inhibitors, such as Bruton tyrosine kinase (BTK) [7–10], phosphoinositide 3-kinase (PI3K) [11], or B-cell Lymphoma 2 (BCL-2) [12, 13], has changed the treatment paradigm by providing even more effective therapeutic options for CLL patients.

Immunotherapeutic strategies (immune checkpoint inhibitors, bi-/tri-specific antibodies, chimeric antigen receptor (CAR)-T-cells) are emerging as relevant treatment options in many malignancies by reverting or bypassing immunosuppression caused by the tumor, but the results have been disappointing so far in CLL. Effective restoration of the immune competence of a patient should have anti-tumor effects that may help control the disease.

**Fig. 1:**
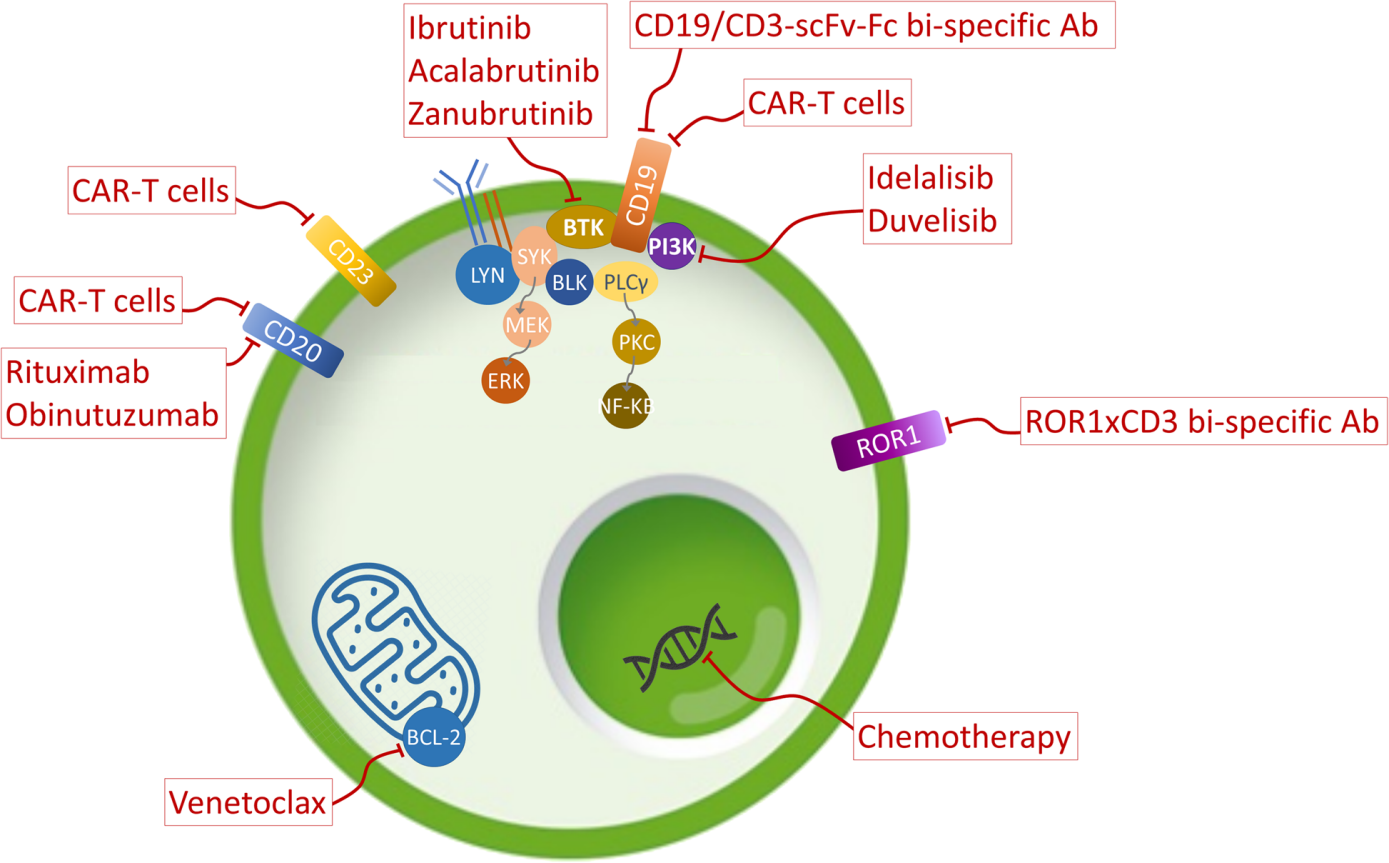
Current relevant therapies in the treatment of patients with CLL that are discussed in this review and main molecular targets. Ab, antibody; BCL-2, B-cell lymphoma 2; BTK, Bruton’s Tyrosine kinase; CAR-T, chimeric antigen receptor T; Fc, fragment crystallizable region; PI3K, phosphoinositide 3-kinase; ROR1, Receptor Tyrosine Kinase Like Orphan Receptor 1; scFv, single-chain variable fragment

There is accumulating evidence of the relevance of the interaction between CLL B-cells and the tumor microenvironment. Restoring autologous T-cell responses remains a potential therapeutic option in CLL, since it should promote anti-tumor immunity without the GvHD (graft-versus-host disease) complications of allogenic transplants [14]. Here, we review the current treatment options (Fig. 1) in CLL in the context of the new molecular understanding of the immune system and its interactions with this neoplasm.

## Pathophysiology of the Immune System in CLL

Immune dysfunction is a fundamental characteristic of CLL that can be present even in early stages of the disease. However, these immunologic aspects have been a deprioritized side in the research of this field for many years. The clinical consequences are an increased risk of secondary malignancies [15] and infections, the latter being one of the leading causes of mortality among patients with CLL [16]. Conversely, in some cases, the perturbation of the immune regulation can result in autoimmune cytopenia in CLL [17, 18]. In this section, we will review the causes and consequences of the immune dysregulation that characterizes CLL.

### Immunosuppressive environment in CLL

CLL B-cells have shown to have immunosuppressive effects on normal T and B-cells by direct cell-to-cell contact and by tumor-derived soluble factors [6].

It has been proposed that CLL B-cells may resemble natural B-reg cells [6] since CLL B-cells also secrete IL-10, which contributes to their immunosuppressive function, and inhibit TNF-α secretion by macrophages. In addition, IL-10 can have autocrine deleterious effects by promoting proliferation and differentiation of CLL B-cells. Clinically relevant, increased IL-10 levels have been associated with a diminished survival in CLL patients [19].

Moreover, a high number of T-reg cells are observed in CLL, which may negatively impact antitumor immunity provided by tumor-infiltrating lymphocytes (TILs) [20].

Myeloid-derived suppressor cells (MDSCs) are more abundant in CLL patients than in healthy individuals. Tumor-associated macrophages (TAMs) secrete immunosuppressive cytokines (IL-10 and TGF-β). Both MDSCs and TAMs promote expansion of T-reg cells and inhibit activation of T-cell surveillance providing a suppressive microenvironment that allows CLL proliferation [21, 22].

The tumor microenvironment is also immunosuppressive in the CLL lymph nodes (LN) with an increased number of tumor-supportive lymphocytes (follicular T helper (Tfh) and T-reg) and fewer cytotoxic ones, compared to peripheral blood. CD8+ T-cells, in addition, have an increased expression of the inhibitory receptors PD-1 that limits their potential to build an immune response in the long term due to stable epigenetic changes difficult to revert [23, 24].

### Defects in the innate and adaptive immune systems

**Fig. 2:**
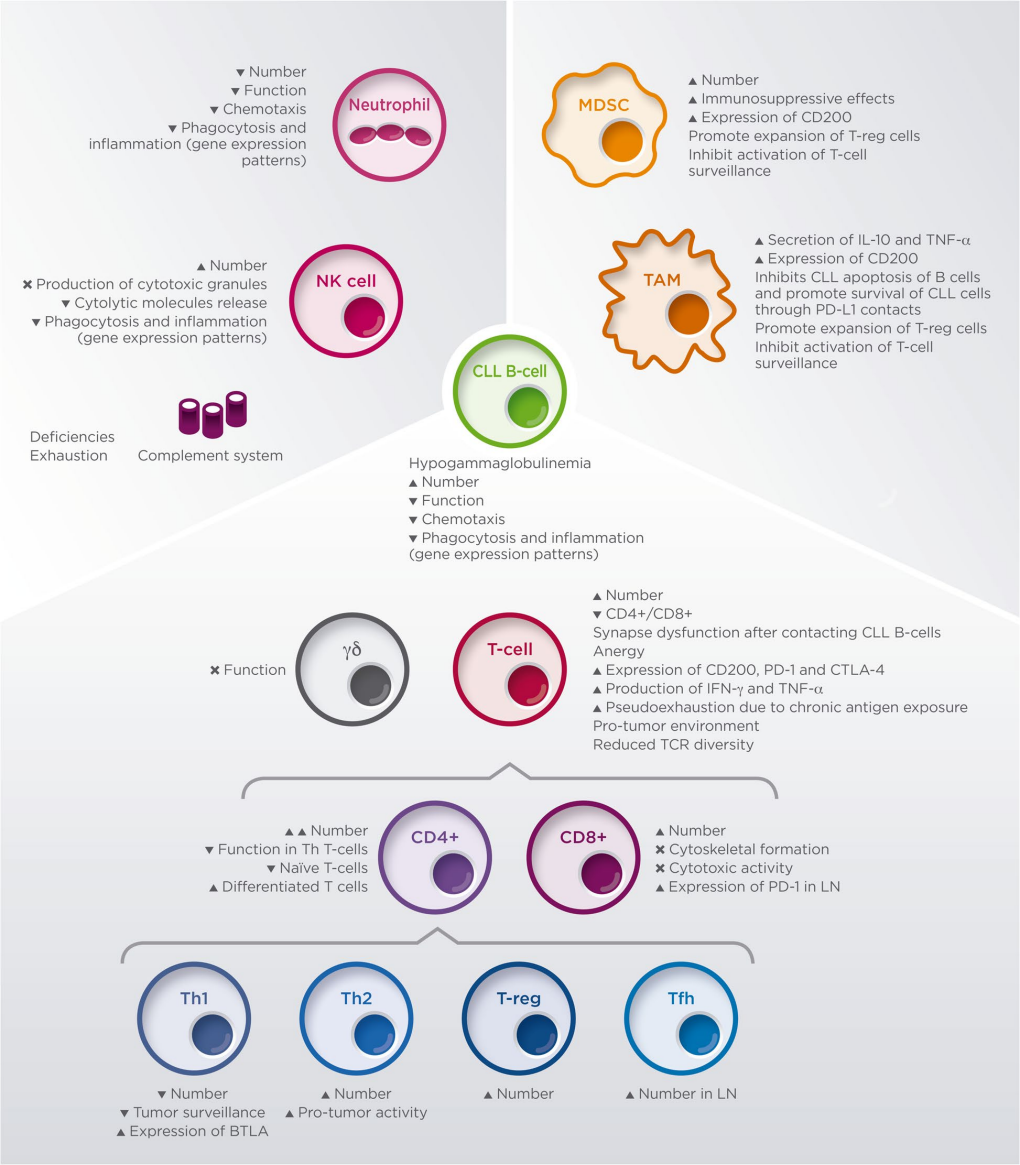
Alterations of the immune system caused by Chronic Lymphocytic Leukemia (CLL) B-cells. BTLA, B- and T-lymphocyte attenuator; CD, cluster of differentiation; CLL, chronic lymphocytic leukemia; CTLA-4, cytotoxic T-lymphocyte-associated protein 4; IFN-γ, interferon gamma; LN, lymph node; MDSC, myeloid-derived suppressor cells; NK, natural killer; TAM, tumor-associated macrophages; PD-1, program cell death protein 1; TCR, T-cell receptor; Th, T helper; TNF-α, tumor necrosis factor alpha; T-reg, regulatory T-cell; X, impairment

Defects in the innate immune system (Fig. 2) have been reported in CLL patients: the phagocytic function and chemotaxis of neutrophils are reduced [25]. Circulating monocytes have gene expression patterns related to deregulation of phagocytosis and inflammation, potentially due to effects of CLL-derived soluble inhibitory molecules [26]. Similarly, NK (natural killer) cells are increased but have impaired production of cytotoxic granules and reduction of cytolytic molecules releases [27]. In addition, low expression of activating molecules in the surface of CLL B-cells adds to the already poor function of NK cells [15].

Adaptive immunity is also dysfunctional, with hypogammaglobulinemia that is exacerbated with disease progression and possibly involving all immunoglobulin classes. In recent studies, a direct correlation has been observed between low levels of IgG and IgA and increased mortality due to bacterial infections [6].

Regarding T-cell immunity, the absolute numbers of CD4+ and CD8+ T-cells are higher in patients with CLL than in age-matched healthy donors but not proportionally higher since the ratio CD4+/CD8+ is reduced. Interestingly, the reduction of CD4+/CD8+ ratio has been shown to correlate with disease progression. Also, the impaired function of the main subsets of T-cells has been described including both CD4+ T helper (Th) cells and CD8+ T cytotoxic lymphocytes cells with defects in cytoskeletal formation and cytotoxic activity [28].

### Impaired anti-tumor T-cell response

The T-cell compartment dysregulation in CLL was described a few decades ago; however, the role of T-cells in the pathogenesis of CLL is still not fully understood. CLL B-cell expansion occurs in parallel to an uncontrolled T-cell proliferation, but it is unclear if the increase is the natural response of the immune system to CLL or if it is an active player of the disease.

Cell to cell communication is also impaired in the CLL environment: synapse dysfunction was identified in T-cells that have been in contact with CLL B-cells inducing T-cell anergy [29]; PD-1+ T-cell frequency is increased in progressive CLL which together with chronic low-affinity self-antigen exposure induces a state of “pseudo-exhaustion” [30].

**Fig. 3:**
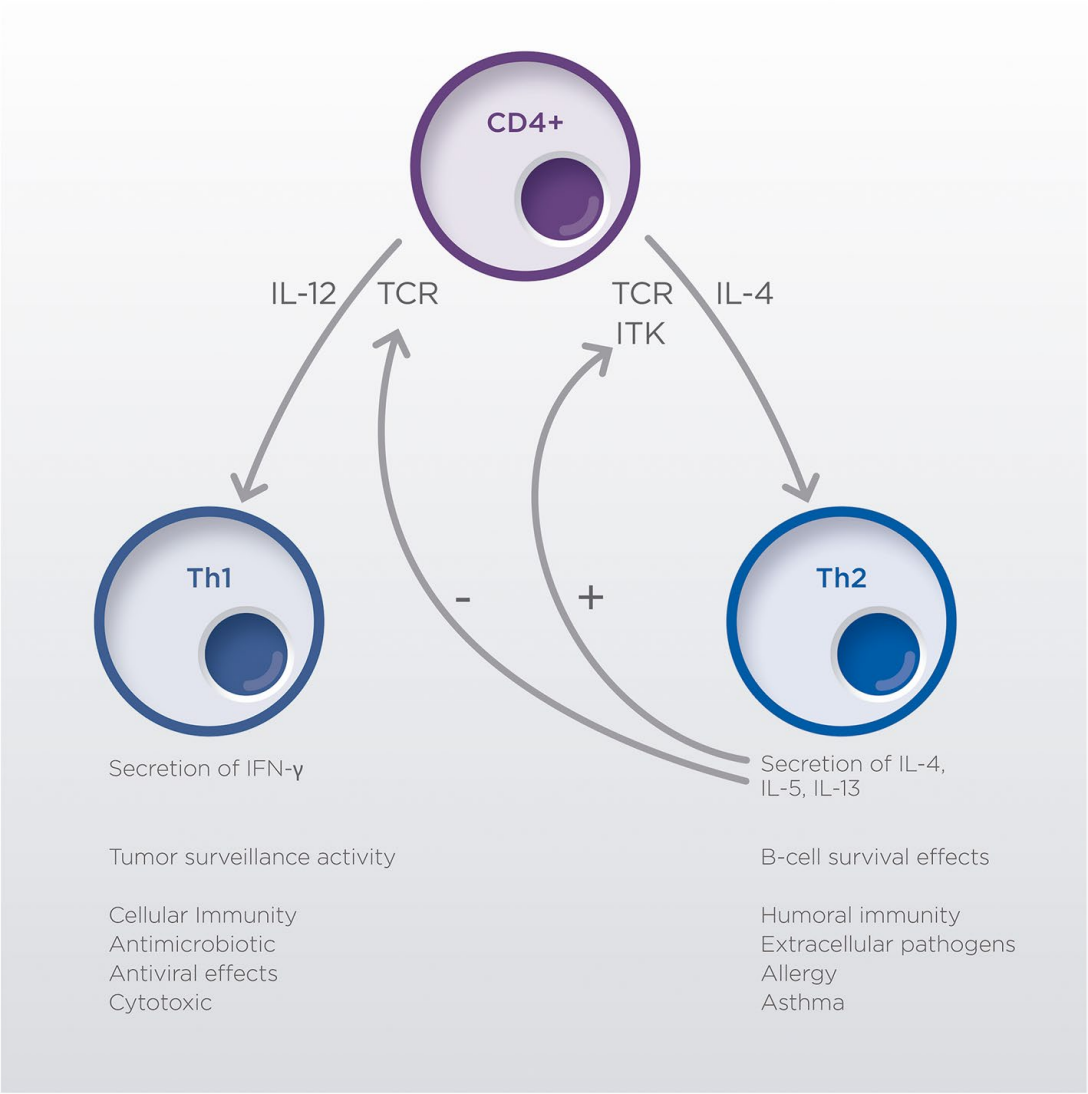
Equilibrium between Th1 and Th2 subgroups of T-cells can impact significantly immune tumor surveillance in CLL. CD, cluster of differentiation; IFN-γ, interferon gamma; IL, interleukin; ITK, interleukin-2-inducible T-cell kinase; TCR, T-cell receptor; Th, T helper

Additional perturbations of T-cell subsets have been described and one of the most interesting is the disruption between Th1 and Th2 balance (Fig. 3). Th1 cells help keep malignant cells under control by the release of molecules such as IL-2 and IFN-γ; on the other hand, Th2 cells protect malignant cells by promoting B-cell antibody production and interfering with cytotoxic T-cells [31]. In healthy conditions, this system helps maintaining normal immune system homeostasis, but it is altered in CLL patients.

Naïve CD4+ T-cells differentiation towards Th1, Th2 or Th17 cells is controlled by interleukine-2-inducible kinase (ITK), among other molecules. T-cell receptor (TCR) activation (especially low affinity interactions with the major histocompatibility complex (MHC) I) triggers a signal cascade activating ITK and promoting Th2 differentiation. Th2 produce cytokines such as IL-4, IL-5 and IL-13, that trigger positive feedback for proliferation of Th2 cells and cross-inhibit other Th lineages. In CLL, the equilibrium is skewed towards Th2 differentiation, inhibiting effective anti-tumor responses and promoting CLL B-cell proliferation [32].

Recent evidence showed the relevance of another subtype of T-cells important for anti-tumor activity: γδ T-cells can identify and kill tumor cells in a MHC-independent manner and recognize a broader spectrum of neoplastic antigens, but these cells are also affected by the immunosuppressive environment of CLL [33].

## Current treatment approaches and impact in the immune function

### Chemoimmunotherapy

Purine analogs such as fludarabine, cladribine, or pentostatin, and alkylating agents such as bendamustine, chlorambucil or cyclophosphamide, interfere with both tumor and healthy dividing cells. As a consequence, chemotherapy reduces CLL B-cell numbers, but also decreases other healthy cells including T-cells and slows down the recovery to normal levels, which ultimately leads to infections [34, 35].

Two frequently used CLL regimens until recently are fludarabine/cyclophosphamide/rituximab (FCR) and bendamustine/rituximab (BR). For many years, FCR has been the standard of care in previously untreated young and fit CLL patients. FCR resulted in high overall response rates with a complete responses of 44%, and median progression-free survival (PFS) of 51.8 months [36]; the benefit was primarily driven by low-risk patients with mutated *IGHV* and without 17p deletion [37]. However, this combination is considered too toxic for frail and/or elderly patients due to appearance of hematological toxicities [36]. Fludarabine can cause reductions in T-cell numbers, with a higher effect on CD4+ cells [34], γδ [34] and T-reg cells [38], and serious opportunistic T-lymphopenia–associated infections during the first year after FCR treatment, such as *Pneumocystis jirovecii* and *Legionella pneumoniae*, pulmonary aspergillosis, endemic fungus infections or disseminated listeriosis [39]. Also, long-term toxicities including risk of secondary hematological malignancies (i.e, acute myeloid leukemia, myelodysplasia) have been associated with the FCR regimen [40]. T-cell repertoire renewal after FCR treatment was found to be affected through ablation and immune reconstitution, rather than expansion of previous T-cell clones, thus impairing early antigen recognition [41].

BR has demonstrated efficacy in CLL although not as significant as FCR [42]. This regimen is frequently used in patients unsuitable for fludarabine-based treatments. It is associated with fewer hematological complications and infections compared FCR, but reductions of CD4+ cells, delays in recovering normal CD4+ cells levels and infections were common [35].

Anti-CD20 antibodies (rituximab and obinutuzumab) target CLL B-cells for complement-dependent cytotoxicity (CDC), direct cell death, antibody-dependent cell-mediated phagocytosis (ADCP) or antibody-dependent cellular cytotoxicity (ADCC) by NK cells. Therefore, even though CLL B-cells express low levels of the necessary ligands to activate NK cytolytic activity, anti-CD20-coated CLL cells are able to trigger an ADCC response [15]. It has been reported that rituximab treatment may reduce humoral response to “recall antigens”, which may be due to the depletion of healthy antibody-producing B-cell clones, although how this may affect the rate of infections or secondary malignancies is not clear yet [43].

Obinutuzumab, a type II humanized anti-CD20, has shown different properties from rituximab, displaying superior ADCC and direct cell death activity, similar ADCP but inferior CDC in vitro. Obinutuzumab caused significant and sustained reductions of CD4+ cells after one infusion. In addition, a reduction of CD8+ cells was also detected but levels were normalized after 6 months [44]. These effects were observed 24-72h post-infusion while depletion of B-cells suffered no significant reduction until later. One more effect in the immune system was the early and sustained reduction in NK cells [44].

Therefore, immune-related risks associated with anti-CD20 therapy are infections, for example, hepatitis B reactivation, which has been described in patients with CLL and other B-cell lymphomas [45].

### Novel Agents

#### BTK inhibitors

BTK belongs to the Tec family of kinases and plays a role in immune cell signaling. BTK is part of the BCR (B-cell receptor) signaling pathway that promotes survival and proliferation of malignant B-cells. BTK inhibitors, including ibrutinib, acalabrutinib and zanubrutinib have shown clinical efficacy in patients with CLL [7, 46–48].

BTK inhibitors produce transient lymphocytosis caused by the mobilization of B-cells from LN, bone marrow and spleen to peripheral blood. They also produce changes in the tumor environment due to a decrease in the expression of immunosuppressive molecules such as PD-L1, IL-10, CD200 or BTLA in CLL B-cells [49, 50].

Interestingly, another related Tec kinase, ITK, is also inhibited by some of these small molecules. ITK is an important component of TCR signaling and promotes Th2, T-reg, and Th9 CD4+ cells differentiation and reduces cytotoxic CD8+ and CD4+ Th1 cells differentiation [31]. This is relevant for tumor control since skewing T-cell differentiation towards a Th2 response would create a pro-tumor environment and cause a decrease of Th1 functions in tumor surveillance. Therefore, the inhibition of ITK also has an impact on the T-cell compartment [31].

**Fig. 4:**
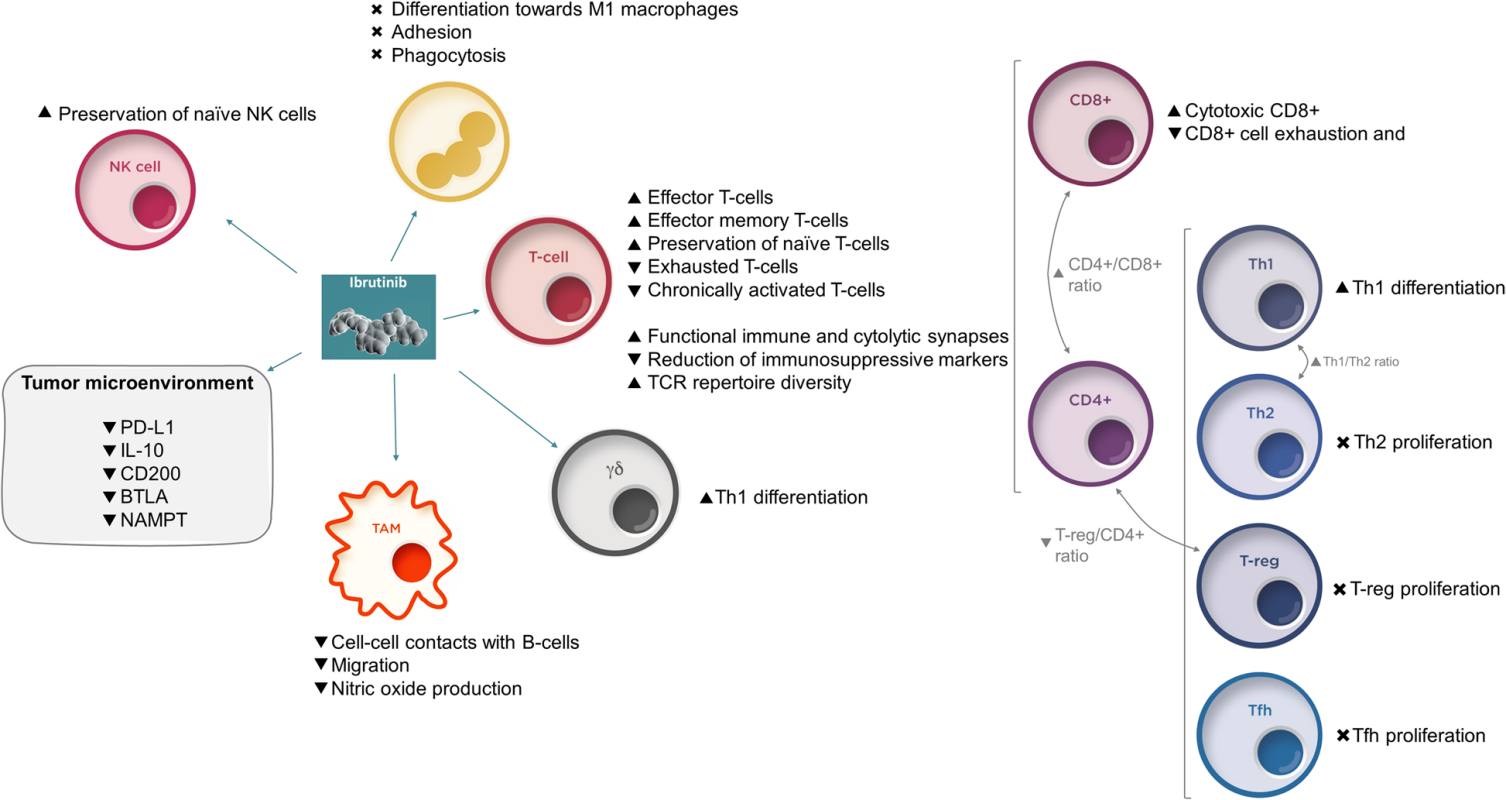
Effects of ibrutinib on immune cells and tumor microenvironment. BTLA, B- and T-lymphocyte attenuator; IL-10, interleukin 10; NAMPT, Nicotinamide Phosphoribosyltransferase; PD-L1, programmed cell death protein ligand 1; TCR, T-cell receptor

Ibrutinib elicits changes in the tumor microenvironment that both control CLL B-cells proliferation and reestablish immune surveillance (Fig. 4). Regarding the T-cell compartment, ibrutinib causes a transient increase of CD4+ and CD8+ T-cells numbers, mostly effector and effector memory T-cells [50], and is associated with an expansion in the TCR repertoire diversity [51]. In the long term, ibrutinib causes a decrease in pathologically high circulating B-cells and exhausted and chronically activated T-cells but preserved naïve T-cells and NK cells [52]. Ibrutinib also shifts the equilibrium of CD4+ T-cells from Th2 and Th17 cells to a Th1 environment by inhibiting ITK, reverses CD8+ cell exhaustion [53] and favors activation of CD8+ cytotoxic T-cells [31]. Moreover, it has been observed that ibrutinib-based therapy improves functional immune and cytolytic synapses between T-cells and CLL B-cells compared to FCR treatment, and enhances the polarization of perforin towards CLL B-cells in T-cell synapses [41], helping to restore the cytotoxic capacity of T-cells to kill tumor cells [54].

In addition, ibrutinib is associated with a the reduction of the T-reg/CD4+ cells ratio, the net increase of Th17 cells and the inhibition of the expression of PD-1 and CTLA-4 in CD4+ and CD8+ T-cells all together leading to a reduction in the immunosuppressive status and an improvement in immune surveillance [50, 55].

Ibrutinib also modulates the function of other immune cells, such as macrophages, by reducing the cell-cell contacts with CLL B-cells in the bone marrow, migration and nitric oxide production [55, 56], and of monocytes, by impairing differentiation towards M1 macrophages, causing adhesion and phagocytic deficiencies and increasing the immunosuppressive profile of nurse-like cells [57].

These changes in the immune system triggered by the treatment with ibrutinib have positive clinical relevance; for example, the restoration of TCR diversity has been shown to correlate with clinical efficacy and lower infection rates [31, 51, 53]; and the restoration of the CD4+/CD8+ ratio may help improve pre-existing autoimmune cytopenias and decrease the rate of emergent events [18].

In summary, ibrutinib effectively controls CLL via a dual mechanism. First, it has shown to act directly on CLL B-cells, reducing the number of malignant cells and their immunosuppressive effects and second, through modulation of the microenvironment and restoration of the physiological immune function, by increasing T-cell-dependent immune surveillance. With the available evidence up to date, ibrutinib is the first therapy to show that a strategy to address tumor burden and immune function simultaneously could improve the disease management (Fig. 4). Moreover, this offers significant potential to combine with other therapeutic options.

Acalabrutinib is a selective inhibitor of BTK that has shown efficacy in CLL [46, 58]. Since ITK binding is essentially absent for acalabrutinib, the effects on Th2 to Th1 switch in the CD4+ T-cell compartment are not observed [50]. Similarly to ibrutinib, a reduction of immunosuppressive molecules such as CD200, BTLA, PD-1 and CTLA-4 expression, together with a decreased production of IL-10, have been noted [50]. Acalabrutinib has some other effects on the immune system, for example, it impairs efficient responses on macrophages (M1 polarization and TNF-α secretion) and neutrophils (phagocytosis, oxidative burst and others), important for antimicrobial infection control [59].

Zanubrutinib is another highly specific BTK inhibitor with low ITK activity that has shown potential in CLL [48]. It has shown significant reductions on PD-1 and CTLA-4 expression and a decrease in T-reg cells; but no changes in Th1/Th2 ratio [60].

#### Pi3K inhibitors

PI3K-δ is mostly expressed in hematopoietic cells and frequently overexpressed in B-cell lymphomas. It plays a major role in B-cell signaling and has also been involved in T-reg cells and MDSC function [11]. Two Pi3K inhibitors have been approved, idelalisib and duvelisib). Idelalisib is a PI3K-δ selective inhibitor with efficacy in CLL by inhibiting CLL B-cell proliferation, survival, adhesion, and homing [11].

Idelalisib has also effects on non-B-cells: it decreases the number of T-reg cells, although this effect was observed mostly in patients with hepatotoxicity [61], and impairs their differentiation and suppressive functions [20]. In addition, it decreases the production of cytokines and other factors such as TNF-α, CD40L and IL-6 by T-cells; IFN-γ by NK cells and IL-10 by T-reg cells [20].

The reduction of T-reg cells and soluble pro-tumor factors makes idelalisib an interesting candidate for combination with immunotherapies but it is not frequently considered an option because those additional effects have been associated with relevant immune-related toxicities of concerning severity and frequency, such as hepatotoxicity or enterocolitis [61].

#### BCL-2 inhibitors

BCL-2 is an anti-apoptotic factor that is overexpressed in CLL. Venetoclax is a BCL-2-selective BH3-mimetic that has been effective in treating patients with CLL, by directly inducing apoptosis of CLL B-cells [12].

Venetoclax has also effects on the T-cell compartment, reducing the total number of CD4+ and CD8+ cells although this is thought to be an indirect effect of the decrease of the tumor. Among the surviving cells, there was a high proportion of CD4+ and CD8+ effector memory T-cells while the proportion of naïve T-cells was lower, these remaining cells kept their proliferation capacity intact [62]. In addition, venetoclax reduces the immunosuppressive environment by reducing the number of Tfh cells, T-reg cells and PD-1+ CD8+ T-cells, as well as the overproduction of inflammatory cytokines [62].

Moreover, some studies suggest venetoclax reduces the number of NK cells but restoration of their function has been observed after treatment [63].

#### Immunotherapies

CAR-T cells are T-cells from a patient that are modified ex vivo and reintroduced to target cancer cells. Infusion of ex-vivo manipulated cells may reduce the influence of the immunosuppressive tumor microenvironment and, conversely, the use of patient’s own cells should reduce the risk of GvHD [14]. Antigens of choice for initial CAR-T cells were the surface antigens CD19, CD20 and CD23, causing also a reduction in healthy B-cells. Overall, either because of lack of efficacy or the development of adverse events, CAR-T cells have not reached clinical expectations in CLL [64, 65], at least partially due to the innate dysfunction in T-cells coming from patients from which the CAR-T cells are produced. Interestingly, therapies that restore immune competency, such as ibrutinib, may enhance the efficacy of CAR-T cells [63]. A similar approach is being studied with CAR-NK cells, and preliminary results suggest better results may be obtained in CLL [66].

Immune Checkpoint Inhibitors (ICI) reactivate immune response towards tumors by blocking inhibitory signals mediated by PD-1 and CTLA-4. ICI have transformed the treatment paradigm in many solid tumors, but their efficacy remains suboptimal in CLL. PD-(L)1 pathway is active in CLL but the expression of PD-L1 in CLL B-cells is usually low. In recent trials, the efficacy of anti-PD-1 monotherapy has been moderate in CLL with Richter transformation (RT), and limited in CLL without RT [67]. Some studies have shown that addition of ibrutinib to the therapy with ICI may improve its efficacy, especially in patients with RT [68, 69].

Another strategy arising in oncology is the development of Bi/Tri-specific antibodies. These molecules are able to force the interaction between tumor and cytotoxic cells and their domains can be combined to target different molecules, for example, CD19, CD20 or ROR1, in B-cells and CD3 in effector T-cells, although other combinations and different effector cells (NK cells) have been used as well [2].

CD19/CD3-scFv-Fc bi-specific antibodies are able to recruit autologous T-cells to kill malignant cells and have shown potential in treating CLL. For example, it has been observed that blinatumomab is able to mediate CLL B-cells and cytotoxic T-cells interaction and trigger tumor cell death in vitro but no activity has been detected in vivo [70]. Interestingly, these effects may be faster in samples of patients treated with some BTK inhibitors [71]. Similarly, ROR1xCD3 bi-specific antibodies had cytotoxic activity against CLL B-cells, but when autologous T-cells were used, only those isolated from patients previously treated with ibrutinib had a significant effect [72].

## Effects of treatment combinations on the CLL immune environment

Considering the potential of the different therapies discussed above on the restoration of the T-cell compartment and other components of the immune system, it is reasonable to try different combinations that, at least theoretically, could improve the patient outcomes by leveraging the patient’s own immune system response (Table 1).

**Table 1:** Available evidence of effects of different therapeutic agents alone or in combination on the immune system in chronic lymphoid leukemia (CLL).

	CIT	BTKi	Pi3Ki	BCL2i	ImmTher
**CIT**	• FCR: T-cells decrease, mostly CD4+, γδ and T-reg • BR: T-cells decrease (CD4+) • C: T-cells decrease • Anti-CD20: complement pathway exhaustion, reduction of humoral response, reduction of CD4+ and transient reduction of CD8+ T-cells, NK cells decrease	• BTK inhibitors can inhibit anti-CD20-induced NK cell cytokine secretion, cell degranulation and FcR-stimulated NK ADCC in vitro • Ibr + anti-CD20: Enhancement of ADCP	• Ide may marginally reduce anti-CD20 effects	• V + Obi: reduction in healthy B-cells, T-cells (Tfh, T-reg and PD-1+ CD8+ T subtypes) and NK cells. Decrease in IFN-γ and TNF-α produced by CD8+ T-cells. Improved NK cell function	
**BTKi**		• Ibr, Aca, Zanu: Decrease of IL-10, CD200 or BTLA in CLL B-cells. Reduce macrophage function, neutrophil to macrophage differentiation. Reduction of PD-1 and CTLA-4 expression in T-cells • Ibr: Transient increase of effector T-cells. Increase in TCR diversity and Th1 cells, activation of CD8+ T-cells. Reduction of exhausted and chronically activated T-cells. Preservation of naïve T-cells and naïve NK cells. Decrease of Th2 and T-reg cells. Improved immune and cytolytic synapses between T and CLL B-cells.		• Ibr+V: reduction in healthy B-cells, T-cells (Tfh, T-reg and PD-1+ CD8+ T subtypes) and NK cells. Decrease in IL-4 by CD4+ cells and TNF-α produced by CD8+ T-cells. Trend of improvement in the antibody production	• Ibr + CAR-T cells: Improved efficacy of CAR-T cells in ibr-treated patients • Ibr + bi-specific antibodies: enhanced activity in Ibr-treated patients • Ibr + ICI: Improved response to anti-PD-L1 treatment, especially patients with RT • br may improve autologous Vγ9Vδ2 T-cell therapy
**Pi3Ki**			• Ide: Decrease of T-reg cells, impairs their differentiation and suppressive functions. Decreases TNF-α, CD40L and IL-6 by T-cells; IFN-γ by NK cells and IL-10 by T-reg cells		• Ide-treated patients showed improved autologous CAR-T cells generation, expansion and cytotoxic effects. • Idelalisib caused a decrease in expression of PD-1
**BCL2i**				• V: reduction of CD4+ and CD8+ cells, increase proportion of effector memory T-cells vs naïve T cells, reduction of Tfh, T-reg and PD1+ CD8+ T-cells. Decreased NK cells but function restored	
**ImmTher**					• CAR-T cells can restore functional capacity of T-cells by in vitro modification • Bi/Tri-specific antibodies may improve interactions between cytotoxic T-cells and CLL B-cells

Aca, acalabrutinib; ADCC: antibody-dependent cellular cytotoxicity; ADCP, antibody-dependent cell-mediated phagocytosis; BCL-2i, B-cell lymphoma 2 inhibitor; BR: bendamustine plus rituximab; BTKi, Bruton’s Tyrosine kinase inhibitor; BTLA, B- and T-lymphocyte attenuator; C, cyclophosphamide; CAR, chimeric antigen receptor; CIT: chemoimmunotherapy; CLL, chronic lymphocytic leukemia; FcR, receptor of antibody fragment crystallizable region; FCR: fludarabine, cyclophosphamide, rituximab; Ibr: ibrutinib; ICI, immune checkpoint inhibitor; Ide: idelalisib; IL, interleukin; ImmTher, immunotherapies; NK, natural killer; Obi: obinutuzumab; PD-1, programmed cell death protein 1; PD-L1, programmed cell death protein ligand 1; Pi3Ki, phosphoinositide 3-kinase inhibitor; T-reg, regulatory T-cell; TCR, T-cell receptor; Tfh, follicular T helper; TNF, tumor necrosis factor; RT, Richter transformation; V, venetoclax; Zanu, zanubrutinib.

### New agents + anti-CD20 antibodies combinations

BTK inhibitors have been studied in combination with anti-CD20 antibodies. BTK inhibitors can inhibit anti-CD20-induced NK cell cytokine secretion, cell degranulation and NK ADCC in vitro [73] but still, these combinations have demonstrated preserved activity of anti-CD20 antibodies clinically. In fact, similar or superior PFS has been observed between acalabrutinib + obinutuzumab vs acalabrutinib alone [58]. Other studies have shown positive synergistic effects when ibrutinib is combined with obinutuzumab [8].

Impact of idelalisib combinations in CLL and specifically in the T-cell compartment has been studied to a lesser extent. Idelalisib has similar effects to ibrutinib when tested in vitro in combination with anti-CD20 antibodies, although inhibition of antibody-dependent cell-mediated effector mechanisms seems to be less pronounced [73].

Studies combining venetoclax and obinutuzumab have shown a decrease not only in CLL B-cells but also healthy B-cells, T-cells and NK cells. Decreases seem to be more profound in Tfh, T-reg and PD-1+ CD8+ T subtypes. In addition, this combination generates a decrease in IFN-γ and TNF-α produced by CD8+ T-cells [23]. In clinical trials, venetoclax plus anti-CD20 antibodies combinations have shown durable responses, in some cases even after cessation of treatment, although the effects of these combinations on the immune function need to be further investigated [74].

### New agents + Immunotherapy

Numerous reports have shown that ibrutinib enhances the effects of immunotherapies. It has been hypothesized [75] that ibrutinib could restore antitumor T-cell immune response in an ITK-dependent manner and enhance ICI effectiveness. A lymphoma model with an ibrutinib-insensitive BTK version has been developed and it has been observed that anti-PD-L1 treatment is T-cell-dependent and moderately effective but the combination with ibrutinib led to significant improvement in the response, through its effects in the immune function restoration rather than the direct effects in CLL B-cells [75]. Two clinical trials have reported data on the combination of nivolumab and ibrutinib. Activity of the combination is limited in CLL patients but is promising in patients with RT [67, 69]. An additional clinical trial reported results on the use of pembrolizumab in CLL and RT patients, where 4 out of 9 patients with RT had objective responses, and none of the 16 patients with CLL. Interestingly, all 4 patients achieving response were previously treated with ibrutinib [68].

An explanation of these results may be that CLL B-cells and RT-cells may be expressing different tumor neoantigens (or RT-cells may be more efficient presenting them). This causes that only RT-cells neoantigens are identified by T-cells, helping ICI trigger responses against them, executed by ibrutinib-mediated re-activated T-cells [67].

Venetoclax helps keep effector memory T-cells viable and functional, and this may be important to improve the effects of immunotherapies [62], although a decrease in naïve T-cells may reduce the response to tumor-derived neoantigens. So far, there is only preliminary in vitro evidence that venetoclax may have synergistic effects with ICI and therefore further research is needed to confirm this observations [62].

### New agents + cell therapies

There is also evidence that ibrutinib can help enhance the CAR-T cell therapy. A preclinical study showed that co-administration of ibrutinib and CD19-directed CAR-T cells (CTL019) in a CLL mouse model improves the efficacy of the CAR-T cells. Ibrutinib can improve the proliferative capacity of T-cells from CLL patients, and it has been shown that the critical expansion of CTL019 is greater when collected from patients that have undergone at least 5 cycles of ibrutinib treatment. In addition, PD-1 and CD200 inhibitory molecules decrease expression in T and CLL B-cells, respectively. Interestingly, these effects have not been observed when cells are treated in vitro with ibrutinib [63]. In clinical trials, ibrutinib-treated patients had significantly better response rates to CAR-T cells compared to ibrutinib-naïve ones, with morphologic complete responses, measurable residual disease negativity, absence of disease in lymph nodes and less severity in cytokine release syndrome [65, 76].

Another type of cell therapy involving another subpopulation of T-cells, Vγ9Vδ2-T-cells, has shown potential in CLL. These cells are able to recognize metabolites produced during malignant transformation and trigger a MHC-independent cytotoxic response [33]. Similar to other T-cells, Vγ9Vδ2-T-cells function is impaired in CLL, and cell expansion in vitro is inefficient. Information regarding this subpopulation of T-cells is limited but preliminary results suggest that ibrutinib may induce Th1 skewing of these cells in vitro, making it a potential combination strategy for autologous Vγ9Vδ2 T-cell therapy [33].

Idelalisib has been shown to improve autologous CAR-T cell generation, expansion and cytotoxic effects when isolated from idelalisib-treated patients or when incubated with idelalisib. It promotes enrichment of naïve-like T-cells and increase of CD8+ cytotoxic T-cells. It has also been observed that idelalisib causes a decrease in expression of PD-1 [77].

BTK inhibitors have also demonstrated enhancement of bi-specific antibody efficacy as previously explained creating an interesting rationale to combine these agents.

### Other combinations

Both ibrutinib and venetoclax have been reported to reduce tumor pro-survival Tfh and T-reg cells and re-activate T-cells as monotherapies [50, 78]. The combination of both molecules has been studied in patients with CLL with results reaching superior complete responses and MRD negativity [79]. Ibrutinib enhances venetoclax killing effects by sensitizing CLL B-cells to the BCL-2 antagonism of venetoclax [80].

Regarding effects in the immune environment, research is still ongoing but there is some data from the combination of venetoclax with ibrutinib or obinutuzumab [23]. With both venetoclax plus ibrutinib or venetoclax plus obinutuzumab strategies, reductions in healthy B-cells, NK cells, and T-cells, specifically a reduction of Tfh-cells, T-reg cells and PD-1+ CD8+ T-cells are observed in vitro [23], mediated mostly by venetoclax.

A decrease in the production of IFN-γ and TNF-α in CD8+ T-cells has only been observed in the venetoclax plus obinutuzumab combination while a decrease in IL-4 production by CD4+ T-cells has only been observed in the venetoclax plus ibrutinib combination, consistent with the restoration of the Th1/Th2 balance described for ibrutinib [23]. Other relevant differences are that an improvement in NK cell function has only been observed in the venetoclax plus obinutuzumab combination while a trend of improvement in the antibody production has only observed in the venetoclax plus ibrutinib combination [23].

CLL is a heterogeneous disease caused by dysregulation at multiple levels. Current treatment options are not curative and complex combinations with multiple drugs are being tested to reduce the residual disease in a durable way and improve anti-tumor response by the immune system. Targeting various disrupted pathways at the same time seems a reasonable option to increase the efficacy and reduce relapses, but careful consideration must be taken because the molecular pathways leading to disease and the off-targets effects are not fully understood. Key factors to consider when designing combination studies are (1) targeting different molecular pathways with scientific evidence of synergistic effects, (2) using treatments with non-overlapping safety profiles, (3) designing the treatment regimen to minimize undesired effects and (4) including a significant set of biomarkers that allows the interpretation of the results.

Examples of such complex regimes are ibrutinib plus obinutuzumab plus venetoclax, designed to maximize the efficacy keeping adverse events low. Positive results have been recently confirmed in a phase 2 trial [81], showing deep responses, and an acceptable safety profile. Various phase 3 cooperative group studies are currently ongoing (NCT04608318, NCT03701282 and NCT03737981). Other combinations still need further investigation.

## Role of the immune system in resistance to treatment

Despite recent progress on treatments for patients with CLL, progressive disease is still frequent in many cases. Due to selective pressure in the presence of therapies, resistant clones may proliferate and cause the disease to progress. Targeted therapies are especially prone to resistance with relatively simple mutations in the target protein or pathway, but thanks to methodological advances, we are starting to understand much more complex signaling networks that can create forms of resistance beyond classic genetics. Single-cell technologies, immune-phenotyping, single-cell transcriptome profiling, functional studies, and chromatin mapping, all integrated with bioinformatic analytic tools, may help understand the supportive role of the immune microenvironment in CLL progression and the co-evolution of cells involved in the disease, directly or indirectly [5, 41, 52, 82–84].

Mutations in BTK and PLCG2, the kinase immediately downstream of BTK, have been described as common mutations in patients progressing on ibrutinib [85, 86]. Interestingly, these resistant clones would provide protection to neighboring cells with wildtype BTK [87], and they were detectable many months before clinical progression [88]. Resistance to ibrutinib can go beyond the BTK pathway, and it has been described for MCL that tumor microenvironment provides initial drug resistance through interactions with the malignant cells through integrin β1-ILK [89] and through multiple other signaling networks in CLL, identified by patient- specific signatures [83].

Similarly, mutations in *BCL2*, detectable months earlier than clinical progression using droplet digital PCR, have been shown to decrease the efficacy of venetoclax [90]. Activated T-cells produce IL-4 and IL-21 that can stimulate the CD40-CD40L interaction and increase the expression of the anti-apoptotic factor BCL-xL, among other factors [91].

One of the most frequently used strategies to avoid proliferation of resistant clones is combining different targeted therapies. Combination of ibrutinib and venetoclax would reduce the probabilities of proliferation of clones with resistant mutations in both pathways simultaneously. Additionally, it has been shown that microenvironment cytokines (IL-10, sCD40L or CpG-ODNs) can significantly reduce the synergy of the combination in an NF-κB-dependent manner [91, 92].

In general, the immune microenvironment creates protective conditions through a network of complex signal interactions to resist therapeutic interventions. Hypoxia has been identified as key condition on setting a protective niche for CLL cells. Under hypoxia, macrophages acquire a tumor-permissive M2 phenotype, T-cells become immunosuppressive and MDSC increase presence [93, 94] directly impacting cell survival and drug resistance. Ibrutinib-induced apoptosis, for example, is reduced under these conditions [95], but at the same time, ibrutinib treatment can counteract, at least partially, the establishment of the protective microenvironment, by suppression of NAMPT (factor involved in linking oxygen use, metabolism, and immune function) transcription, important for the differentiation of tumor-supporting M2 macrophages [96]. A key mediator of the hypoxia effects is HIF-1, found to be overexpressed in CLL [95] and it has been shown that inhibition of HIF-1 with BAY87-2243 has synergistic effects when combined with other agents, such as ibrutinib [95].

Interestingly, hypothesis-generating studies have shown that impairing the microenvironment can help improve efficacy of targeted therapies in general. Ascorbic acid is a pro-oxidant agent that has shown to overcome the protective effect of the microenvironment. It can potentiate the effects of ibrutinib, idelalisib and venetoclax in vitro and it was able to reduce the viability of CD40L/IL-4- stimulated CLL cells [97].

Even though these results are preliminary, new high throughput and single-cell methods may help understand better the complexity of these interactions and can contribute to design therapeutic combination strategies that target the tumor microenvironment to reduce progression in CLL and identify patterns that are able to predict response to treatment.

## Clinical considerations of immune restoration/Expert insight

Patients with CLL have a characteristic disruption of the immune function and addressing it in parallel to the tumor burden should be central in the management of the disease. Treating the disease by restoring the anti-tumor activity of the patient’s immune system can lead to sustained and deep responses and reduce side effects and non-progression deaths.

CLL treatments may exacerbate immune defects, causing infections, autoimmune diseases or secondary neoplasias [98]. Data on these effects are scarce but we are starting to understand the impact of CLL treatments on these side effects:

### Infections

Chemoimmunotherapy effects on the T-cell compartment may also lead to infections [16]. Idelalisib has been associated with an increase in the risk of *Pneumocystis jirovecii* pneumonia and other opportunistic infections [61]. ICIs have shown multiple examples of immune-related adverse events in different tumors, requiring immunosuppressive treatment leading to secondary infections [98]. Blinatumomab causes hypogammaglobulinemia and venetoclax has been associated with neutropenia and infections [98]. A slight increase in bacterial, viral and opportunistic infections has been observed with ibrutinib, caused by defects in the innate immune function, although these infections appear mostly during the first months of therapy. Interestingly, prolonged treatment with ibrutinib restores humoral immunity and immunoglobulin levels [98] and a reduction of infections has been reported when ibrutinib is used beyond 6 months [99]. Moreover, ibrutinib may also attenuate the exacerbated immune response in severe infections, such as in the case of visceral leishmaniasis [100] or *Staphylococcus aureus* [101], or reduce the burden of the inflammatory process during the infection, for example in the case of COVID-19 [102] or pneumococcal pneumonia [103].

### Autoimmune cytopenias

Chemoimmunotherapy may impair further the function of effector T-cells, and in some cases, such as fludarabine-based regimens, may increase the risk of autoimmune cytopenias (AIC), although addition of cyclophosphamide appears to decrease the incidence of fludarabine-dependent AIC [17]. Idelalisib has shown to reduce T-reg cells, which are key players in the control of autoimmune responses and excessive reactions to non-self-antigens [61]. In the case of ibrutinib, data have shown that it causes a reduction of Th17 cells, decreasing the risk of treatment-emergent AICs [17], and providing better control in patients with pre-existing AIC [18].

### Secondary malignancies

The incidence of secondary malignancies is increased in CLL patients potentially due to the disrupted immune surveillance. CIT regimens such as FCR have been associated with a higher incidence of secondary malignancies, particularly hematological neoplasias [40]. This effect has been shown to be exacerbated in patients with prolonged cytopenias, potentially due to the prolonged treatment with rituximab. Recently, in a retrospective unicentric study of patients treated with BTKi in first-line (20% of patients) and in the relapse/refractory setting (60% of patients received alkylating agents), the observed rate of secondary malignancies was 2.2, similar to the reported rate in a large cohort of CLL patients followed at a single tertiary center prior to the use of BTKi [104–106]. In the CLL14 trial, secondary malignancies seem more frequent in venetoclax combined with obinutuzumab (18%) vs. chlorambucil plus obinutuzumab (10.3%) after a follow-up of 3 years. Additional follow-up will be needed to confirm this association [13, 107].

## Conclusions

CLL has undergone relevant clinical advances in the past decades, both in the understanding and the treatment of the disease. For many years, the focus of the therapeutic approaches has been to decrease the number of B-cells to reduce the tumor burden. New insights have shown that the interaction of leukemic cells with T-cells and other players of the immune system participate in the pathogenesis of the disease and contribute to a decrease of the overall health status of the patient, but this knowledge has not fully been applied to clinical practice yet. Some real-world studies are starting to look at relevant immune parameters, such as IgA in ibrutinib-treated patients [108] but more information is needed to have a broader picture of the dynamics of the whole immune system in the treated patients. We need to further understand the roles of the different immune cells in the disease to apply therapeutic strategies that not only eliminate malignant B-cells but also restore the immune competence of the patient toward anti-tumor surveillance and immunity.

There is growing evidence that T-cells play a very relevant role in the disease progression and understanding how T- and B-cells create a pro-tumor environment is essential to treat CLL patients. CLL B-cells can cause an immunosuppressive effect on T-cells, which exhibit exhaustion features that abrogate immune control of the tumor. A whole pro-tumor strategy is put in place by the immune system: decrease in Th1 cells (anti-tumor) is offset by an increase of Th2 cells (pro-tumor), cytotoxic T-cells number and function are inhibited, immunosuppressive MDSC, T-reg cells and Tfh cells increase their function, inhibitory cytokines (i.e. IL-10) and checkpoint receptors (i.e. PD-1) are upregulated and the conditions in the lymph nodes are even more immunosuppressive.

Keeping in mind that not only eliminating malignant B-cells, but also restoring the immune function may have significant positive consequences in the clinical practice, especially in CLL, that immune dysfunction is the cause of most of the complications for this disease. Also, understanding the relevance of the other immune players in CLL and how different treatments affect them would have a direct impact in successfully managing the disease. First, because this will allow us to avoid therapies that negatively impact the immune function, such as some chemotherapies. Second, because it will reduce the number of adverse events related to the immune dysfunction, for example infections, autoimmunity and second malignancies. Third, because this will facilitate the design of combination strategies, not only to reach deeper durable responses but to attack the disease from different fronts, with higher chances of synergistic and long-term effects. And four, it will allow us to successfully use the new generation of therapeutic strategies, most importantly immunotherapies and cell therapies, in CLL, so far inefficacious due to the immune dysfunction in CLL patients.

Management of CLL multilaterally may be the best option for patients but still, caution with unexpected effects, and prevention of known adverse events together with prompt management of side effects may help in the practice until a better understanding of the disease and the effects of the different treatments is achieved.

There is still need for more research to understand the effects of each treatment option (monotherapies and combinations) in the tumor environment and current technology can yield insightful data. Thus, it is essential to include broader biomarker analysis in clinical studies, that go beyond analysis of B-cells and include T-cell subpopulations and soluble factors at baseline and different timepoints.

Moreover, whatever we learn about immunotherapies in such a paradigmatic immunosuppressed disease as CLL can help improve their efficacy in other tumors where immune function is impaired.

## Data Availability

Not applicable

## Abbreviations

ADCC: antibody-dependent cellular cytotoxicity
ADCP: antibody-dependent cell-mediated phagocytosis
AIC: autoimmune cytopenias
BCL-2: B-cell Lymphoma 2
BCR: B-cell receptor
BR: bendamustine/rituximab
BTK: Bruton tyrosine kinase
CAR: chimeric antigen receptor
CDC: complement-dependent cytotoxicity
CIT: Chemo(immuno)therapies
CLL: Chronic Lymphocytic Leukemia
CpG-ODNs: CpG oligodeoxynucleotides
CTL019: CD19-directed CAR-T cells
FCR: fludarabine/cyclophosphamide/rituximab
GvHD: graft-versus-host disease
HCK: hematopoietic cell kinase
ICI: Immune Checkpoint Inhibitors
ILK: integrin-linked kinase
ITK: interleukine-2-inducible kinase
LN: lymph node
MCL: mantle cell lymphoma
MDSC: myeloid-derived suppressor cells
MHC: major histocompatibility complex
NAMPT: nicotinamide phosphoribosyltransferase
NF-κB: nuclear factor kappa-light-chain-enhancer of activated B cells
NK: natural killer
PFS: progression-free survival
PI3K: phosphoinositide 3-kinase
PLCG2: phospholipase C gamma 2
RT: Richter transformation
T-reg: T regulatory cells
TAM: Tumor-associated macrophages
TCR: T-cell receptor
Tfh: follicular T helper
Th: T helper
TILs: tumor-infiltrating lymphocytes
